# Humeral Septal Aperture in Ancient Tombos Nubians

**DOI:** 10.1155/tswj/5556047

**Published:** 2026-02-19

**Authors:** Jenessa Love, Ashlyn J. Morris, Heidi Joelle Althaus, Faith Kylee Darden, Michele R. Buzon, John E. Kuhn, Randall T. Loder

**Affiliations:** ^1^ Department of Anthropology, Purdue University, West Lafayette, Indiana, USA, purdue.edu; ^2^ Office of Student Affairs, Indiana University School of Medicine, Indianapolis, Indiana, USA, indiana.edu; ^3^ Department of Orthopaedic Surgery, Vanderbilt University School of Medicine, Nashville, Tennessee, USA, vanderbilt.edu; ^4^ Department of Orthopaedic Surgery, Indiana University School of Medicine, Indianapolis, Indiana, USA, indiana.edu

**Keywords:** bilaterality, bioarchaeology, etiology, genetic, metrics, septal aperture, Tombos

## Abstract

**Objective:**

The majority of the published literature regarding the septal aperture (SA) dates to the last 200 years. The archeological literature is sparse. The collection from Tombos along the Nile River (1400–656 BCE) provides an opportunity for further study of SA in ancient populations. The purpose of this study was to determine the prevalence and shape of SA in the population as well as the size of the humerus and correlate that with and without SA in the Tombos population.

**Methods:**

Adult humerus specimens from the Tombos skeletal population with intact distal humeri were studied using both photography and caliper measurements. The caliper and photographic methods gave equal results.

**Results:**

There were 164 distal humeri; an SA was present in 40.9%. There were no differences by sex, age group, or laterality. Of the 34 individuals with bilateral intact paired humeri, 47% had SAs. The involvement was bilateral in 13, left in 2, and right in 1. The shapes of the apertures were oval/elliptical in 73%, irregular in 15%, and circular in 12%. Those humeri with an SA had smaller epicondylar breadths, vertical humeral head diameters, humeral shaft diameters, condylar articular width, and trochlear articular width. There were no differences in the coronoid or olecranon fossa width/height. The 41% prevalence of SA in the Tombos population is similar to the 45%–60% in other African archeological studies but higher than the 20%–37% in prehistoric Native Americans. Smaller humeri had a higher prevalence of SA similar to several modern‐day studies.

**Conclusions:**

The etiology of SA is not definitively known, with mechanical, structural, and genetic etiologies postulated. A high prevalence of bilaterality is supportive of a genetic etiology; our 81% prevalence of bilaterality suggests a strong genetic component in the etiology of SA in this particular population.

## 1. Introduction

There is an extensive body of anthropological literature regarding the distal humeral septal aperture (SA) [1]. A 2019 systematic review of SA found 61 unique manuscripts. While SAs are well known to anthropologists, most of the published literature involves specimens dated to within the last 200 years. The literature from specimens older than 200 years is sparse. We found 19 studies from over 200 years ago (Table 1). A few of the specimens could not be accurately dated and were termed prehistoric [18, 19] and involved Native American populations. The remainder of the studies gave reasonable dates, ranging from the Neanderthal era to the medieval period. Of these studies, only six [2–7] are from the BCE era (Table 1).

**Table 1:** Septal aperture studies.

Author	Reference	Geographic location	Population/ethnicity studied	Time span	Septal aperture prevalence (%)
Smith	[2]	Krapina, Yugoslovia	Neanderthal	7700 BCE or older	60.0%
Baker	[3]	Windover and Indian Knoll, United States	Native Americans	7100–1000 BCE	44.3%
Hrdlička	[4]	Egypt	Egyptian adult	1938–1759 BCE	44.8%
Hrdlička	[4]	Egypt	Egyptian child	1938–1759 BCE	37.7%
Riesle	[5]	Konamazan, Luristan, Iran	Mideastern	1550–1200 BCE	37.5%
Taxman	[6]	Ohio River Valley/Kentucky, United States	Native Americans	300 BCE–200 CE	32.7%
Nagar	[7]	Israel	Multiple	625 BCE–1450 CE	26.0%
Hrdlička	[4]	Karga Oasis, Egypt	Egyptian	200–299 CE	55.2%
Baker	[3]	El‐Hesa cemetery, Egypt	Nubian	350 CE	59.5%
Nagar	[8]	Negev	NA	395–1453 CE	20.6%
Anderson	[9]	United Kingdom	European	410–1450 CE	12.2%
Glanville	[10]	Africans (Tellem, Mali)	African	476–1450 CE	47.0%
Glanville	[10]	Netherlands	European	476–1450 CE	6.1%
Woo	[11]	China	Chinese	581–899 CE	11.1%
Pietrusewsky	[12]	Hawaii	Hawaiian	600–1800 CE	29.0%
Oláh	[13]	Sárrétudvari‐Hízóföld cemetery, Hungary	European	900–999 CE	15.7%
Mays	[14]	Wharram Percy, England	European	1000–1399 CE	6.9%
Myszka	[15]	Ostrów Lednicki, Poland	European	1000–1199 CE	44.0%
Kubicka	[16]	Ostrów Lenicki, Poland	European	1000–1199 CE	45.8%
Myszka	[17]	Cedynia, Poland	European	1000–1399 CE	7.5%
Nagar	[7]	Israel	Arabic	1250–1922 CE	23.9%
Nagar	[8]	Amizaya, Israel	Arabic	1600–1999 CE	40.0%
Lamb	[18]	Arizona, Salado I	Native American	Prehistoric	54.0%
Lamb	[18]	Zuni, New Mexico, Seven Cities of Cibola	Native American	Prehistoric	19.7%
Lamb	[18]	Multiple mounds Midwest and Eastern United States	Native American	Prehistoric	27.4%
Ferguson	[19]	New York	Native American	Prehistoric	20.4%
Ferguson	[19]	New Jersey	Native American	Prehistoric	21.4%
Ferguson	[19]	Maryland, Piscataway	Native American	Prehistoric	26.1%
Ferguson	[19]	Maryland, Port Tobacco	Native American	Prehistoric	32.6%
Ferguson	[19]	Maryland, Anacostia	Native American	Prehistoric	32.2%
Ferguson	[19]	Maryland, Accokeek	Native American	Prehistoric	37.2%
Ferguson	[19]	Virginia, Potomac Creek	Native American	Prehistoric	20.2%
Ferguson	[19]	Virginia, Western	Native American	Prehistoric	19.7%
Ferguson	[19]	Kentucky, Indian Knoll	Native American	Prehistoric	26.3%
McAlister	[20]	Libya	Egyptian	Ancient	57.2%

*Note:* Prehistoric: prior to historical dates. Ancient: old but without dates.

The etiology of SA is unknown, with mechanical, genetic, environmental, and a combination of factors being invoked [4–7, 9, 10, 13, 14, 16, 17, 20, 21]. Mays [14] stated that SAs were more common in females and in left bones, leading early investigators to suggest that they were more frequent in “weaker” bones. However, subsequent literature offered little support for the concept that a relationship between humeral gracility and SA exists in humans beyond any relationships with sex and laterality.

The collection of ancient adult skeletons from Tombos site (in modern‐day Sudan) of the people living along the Nile River during the New Kingdom (1400–1070 BC) and early Napatan (1070–656 BC) periods [22] is an opportunity to garnish additional information regarding the SA in ancient populations and assist in answering the causation question proposed by Mays [14]. While there is one study of SAs in Nubians [3], those individuals lived much later (~350 AD) than the Tombos population (1400–656 BC) [23]. It was the purpose of this study to investigate SA in the Tombos population. The Tombos collection also affords the ability to study potential differences in SA prevalence between two different populations living in the same geographical area, before and after significant sociopolitical changes. Tombos is the site of a community that was established during the New Kingdom colonial period, in which Egyptian immigrants settled in Nubia and interacted with the local communities. After the fall of the Egyptian empire, descendants of the immigrant Egyptians and local individuals continued to thrive in the subsequent early Napatan period when Egypt no longer controlled Nubia [23]. Such information will (1) assist in answering the humeral robusticity question, (2) add to the body of literature regarding SA in ancient populations, and (3) thus lead to a better understanding of its etiology. Specifically, the aims of this study were to determine the prevalence of SA, and when an SA was present, the shape of the SA, the dimensions of the SA, and correlate/compare these with measurements of the humeri in those with and without SA.

## 2. Material and Methods

All adult humerus specimens (240) from the Tombos skeletal population housed at the Department of Anthropology at Purdue University were reviewed to find those with intact distal humeri. Acceptable quality was defined as when the most medial and lateral portions of the distal humerus were intact, with 164 fitting these criteria. The Sudanese National Corporation for Antiquities and Museums permitted the excavation and exportation of human remains. As these specimens studied are from the BCE, informed consent is impossible, and Institutional Board Review approval did not apply, as per Purdue University, the curator of the specimens.

Of the 164 total specimens, 111 were from the New Kingdom and 50 from the early Napatan periods (inconclusive in 3) (Table 2). There were 123 specimens from individual burials and 41 from comingled burials. Of the 123 individual specimen burials, 57 were bilateral, six right, and three left humeri. Of the 41 comingled burial specimens, 22 were the right and 19 the left humerus. The humeri were complete in 121 and incomplete (only intact distal portion) in 43 specimens. Sex could be determined in 118, with 55 male and 63 female specimens. Age could be determined in 116 and was 15–24 years in 16, 25–34 years in 38, 35–49 years in 27, 50–69 years in 23, and 70 years in 12 specimens.

**Table 2:** Categorical variable findings in those with and without a septal aperture.

	All (*n* = 164)	Septal aperture present (*n* = 67)	%	Septal aperture absent (*n* = 97)	%	*p* value
		*n*		*n*		
All	164	67	40.9	97	59.1	
Period						
New Kingdom (1400–1070 BC)	111	41	36.9	70	63.1	0.30
Early Napatan (1070–656 BC)	50	23	46.0	27	54.0	
Sex						
Male	51	17	33.3	31	60.8	0.085
Female	62	32	51.6	29	46.8	
Age group (years)						
15–24	16	10	62.5	6	37.5	0.27
25–34	39	17	43.6	21	53.8	
35–49	28	8	28.6	19	67.9	
50–69	25	11	44.0	12	48.0	
70+	12	4	33.3	8	66.7	
Laterality						
Right	83	32	38.6	51	61.4	0.63
Left	81	35	43.2	46	56.8	
Burial						
Individual	123	51	41.5	72	58.5	0.86
Commingled	41	16	39.0	25	61.0	
Humerus status						
Complete	121	51	42.1	70	57.9	0.59
Incomplete	43	16	37.2	27	62.8	

There were 67 total specimens where an SA was present; 51 were from individual burials and 16 from comingled burials. Of the 51 individual burials, 49 were bilateral and two right and no left humeri. Of the 16 comingled burials, there were eight right and eight left humeri. The humeri were complete in 51 and incomplete (only intact distal portion) in 16 specimens. Sex could be determined in 50, with 18 male and 32 female specimens. Age could be determined in 50 and was 15–24 years in 10, 25–34 years in 17, 35–49 years in 8, 50–69 years in 11, and ≥ 70 years in four specimens.

Digital photographs of the specimens from both anterior and posterior projections were taken with the camera (Nikon D5200 DX format with an AF‐S Nikkor 18–55 mm f3.5–5.6 zoom lens) positioned directly perpendicular to the table and mounted on a copy stand; the specimen was lying flat on the copy stand surface. The camera lens was centered perpendicular to (as determined by a Stanley line and surface bubble level) and directly over the coronoid fossa for the anterior view and the olecranon fossa for the posterior view. The camera anchor bolt was 50 cm above the copy stand platform, placing the front of the lens 38 cm from the copy stand surface. The photographs were taken at a focal length of 48–55 mm (short telephoto range). This telephoto range minimizes parallax due to the longer distance from the camera to the specimen. A standard archeological measurement scale (Strati Concept photographic scale) was placed close to the specimen from which length measurements could be determined.

The photographs were analyzed using two different methods. The first analysis was the determination of an SA present or absent using the criteria of Baker et al. [3]. A true SA has smooth contours throughout its margin; taphonomic damage is typically demonstrated by rough or jagged edges as previously defined [3]. The SA was classified as round, oval, triangular, or irregular as defined by Shivaleela et al. [24] and Pires et al. [1]. The photographs were reviewed by two of the anthropology coauthors. When there was ambiguity regarding the presence of an SA, it was designated as not present to give a conservative estimate regarding prevalence. The ambiguity determination was made by the senior anthropologist (MRB). Laterality was recorded for all specimens, as was the individual’s sex and age when known for the discretely buried individuals using standard osteoarchaeological methods [25–27]. Sex was estimated using methods defined by Buikstra and Ubelaker [26], which include os coxae morphology (ventral arc, subpubic concavity, and greater sciatic notch) and cranial morphology (nuchal crest, mastoid process, supraorbital margin, glabella, and mental eminence). The age at death of the individual was categorized into groups: 15–24, 25–34, 35–49, 50–69, and ≥ 70 years old. Transition analysis (ABDOU Ver 2.1.046 2016) was used to produce maximum likelihood age estimates; this program combines cranial sutures, features of the pubic symphysis, and iliac auricular surfaces in addition to estimated sex and ancestry group [25].

**Figure 2:**
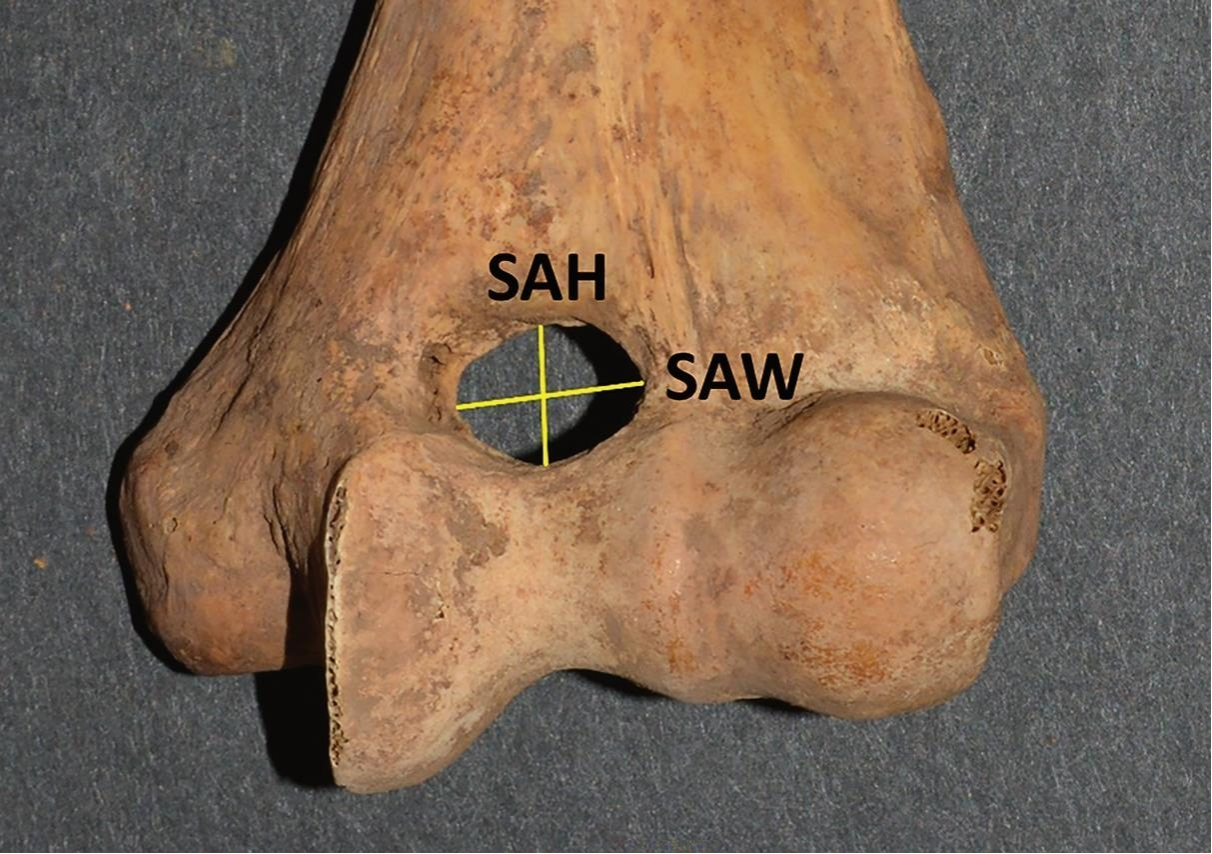
The lines measured from an actual specimen denoting the SA width (SAW) and the SA height (SAH).

The second portion of the analysis involved multiple measurements of the distal humerus from the photographs. As the distal humeri of these specimens were extremely fragile due to their age, photographs were used to make measurements of the SAs. There was a fear of damaging the specimen and the olecranon/coronoid fossa if standard calibers were placed inside these small openings, rendering the specimens unavailable for further study due to such taphonomic injury. The measurements from the anterior photograph were the total distal humeral width (TW), total condylar width (TC), trochlear width distance (TRD), coronoid fossa width (CFW), and coronoid fossa height (CFH). The TW was defined as the distance from the most medial and most lateral points of the epicondyles (TW); the TC is from the most medial to lateral points of the condyles at the joint surface (TC), the TRD as the distance from the medial trochlear ridge to the trochleo‐capitular groove, the CFW as the maximum width of the coronoid fossa, and CFH as the maximum height of the coronoid fossa. From the posterior photographs, the olecranon fossa width (OFW) and olecranon fossa height (OFH), defined as the maximum heights and widths of the olecranon fossa, respectively, were measured (Figure 1). When an SA was present, then the septal height (SH) and width (SW) were measured from both anterior and posterior views (SHA, SWA, SHP, and SWP) (Figure 2).

Figure 1(a) The lines measured from an actual anterior view photograph of a specimen denoting the total width (TW), the total condylar width (TC), the trochlear width distance (TRD), coronoid fossa width (CFW), and coronoid fossa height (CFH). (b) The lines measured from an actual posterior view photograph of a specimen denoting the total width (TWP), olecranon fossa width (OFW), and olecranon fossa height (OFH).

**Figure:**
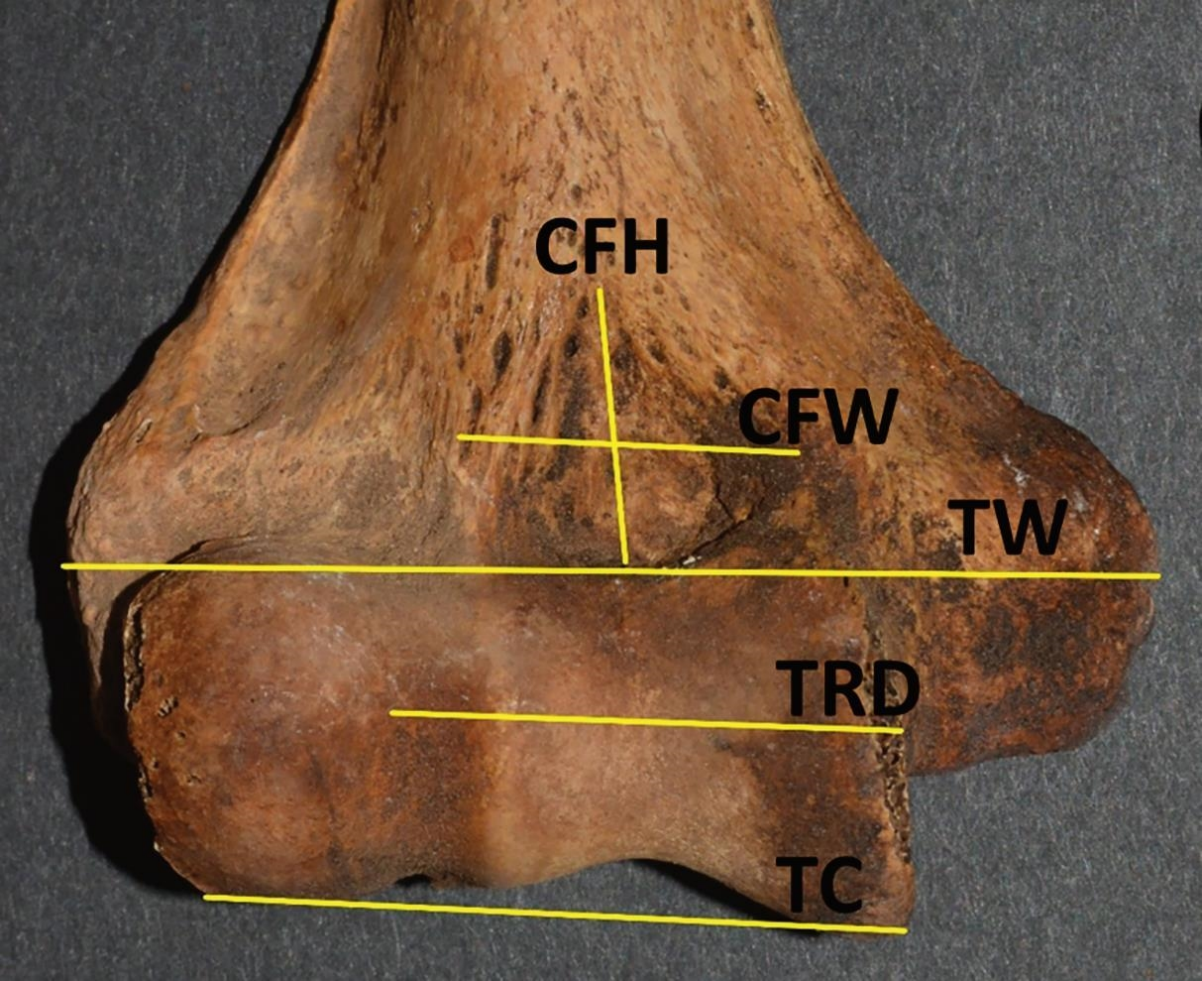
(a)

**Figure:**
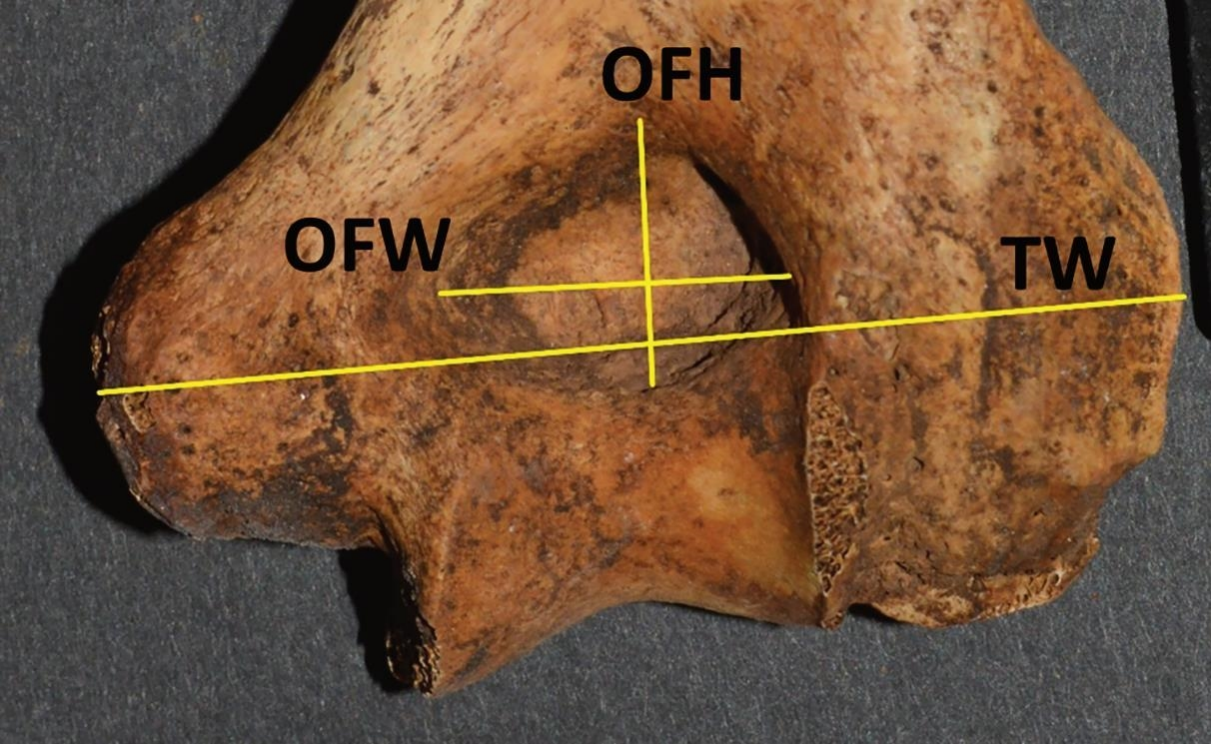
(b)

All measurements were performed with ImageJ software. ImageJ was developed by the National Institutes of Health and is in the public domain (https://imagej.net/ij/index.html). The 10 cm archeological scale placed directly next to the specimen was used to calibrate the ImageJ software to convert the pixel length of the lines to centimeters. The straight line measurement tool in ImageJ was used to measure the length of each line. These measurements were determined from the photographs rather than subjecting the specimens to further caliper manipulation, as we did not wish to risk any more damage to these fragile bones. A previous study [28] has shown that the average difference between caliper and photographic measurements using ImageJ is 0.061 mm.

To analyze bone robusticity, measurements were made on the actual specimen using standard anthropological calipers [26] (Mitutoyo Digital Sliding Calipers) and an osteometric board. These were the humeral length (most superior point on head to most inferior point on the trochlea), maximum vertical humeral head diameter (distance between most superior and inferior points on the border of the articular surface), maximum width of the humerus at the midshaft in both anteroposterior (AP) and medial/lateral (ML) planes, and epicondylar breadth (distance of most laterally protruding point on the lateral epicondyle from the corresponding projection on the medial epicondyle) [26].

### 2.1. Statistical Analysis

Differences between categorical variables were analyzed using the Fisher exact test (2 × 2 analyses) or the Pearson chi‐square test (>2 × 2 analyses). Differences between the two groups of continuous variables were analyzed with the Student’s *t*‐test or ANOVA (three or more groups). Correlations between the SA width and height and the six humeral measurements were analyzed with the Pearson correlation coefficient. Systat 13 software was used for statistical analyses.

## 3. Results

There were 164 distal humeri that were of acceptable quality. An SA was present in 67 (40.9%) and absent in 97 (59.1%) humeri. There were 32 (48%) right humeri with an SA and 35 (52%) left humeri with an SA. There were no differences by time period (New Kingdom vs. early Napatan), sex, age group, right versus left humeri, individual versus commingled burial, and complete versus incomplete humeri (Table 2). There were 34 individuals with paired humeri, both being intact, allowing for a strict right/left comparison. Of these 34 individuals, 16 (47%) had 29 SAs. The involvement was bilateral in 13, the left humerus in two, and the right humerus in one individual. This high percentage of bilaterally (81%) was statistically significant (one‐way Pearson chi‐square of bilateral vs. unilateral, *p* = 0.012). The shapes of the apertures were oval/elliptical in 49 (73%), irregular in 10 (15%), and circular in 8 (12%) (Figure 3).

Figure 3Examples of (a) an oval/elliptical SA, (b) an irregular SA, and (c) a circular SA.

**Figure:**
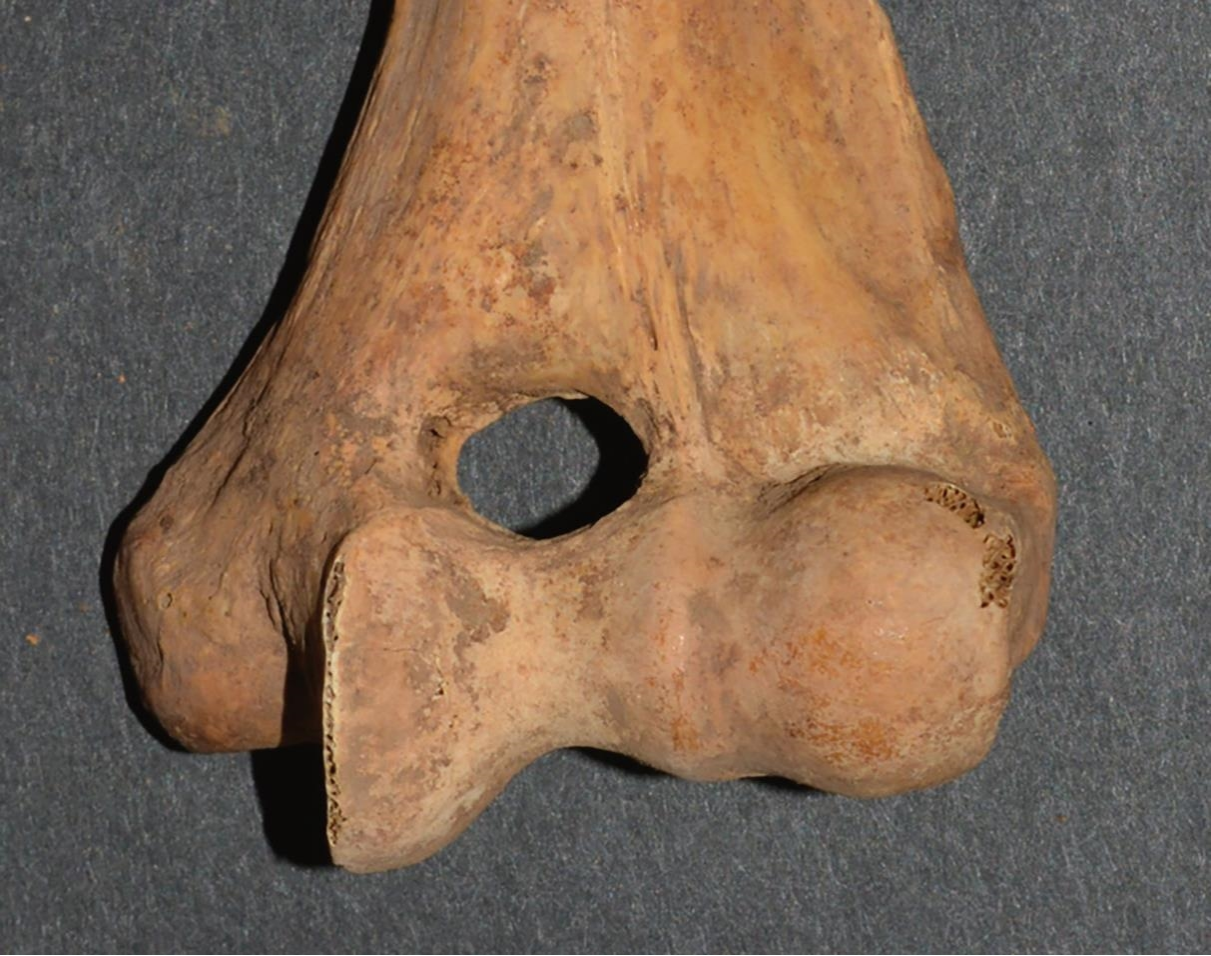
(a)

**Figure:**
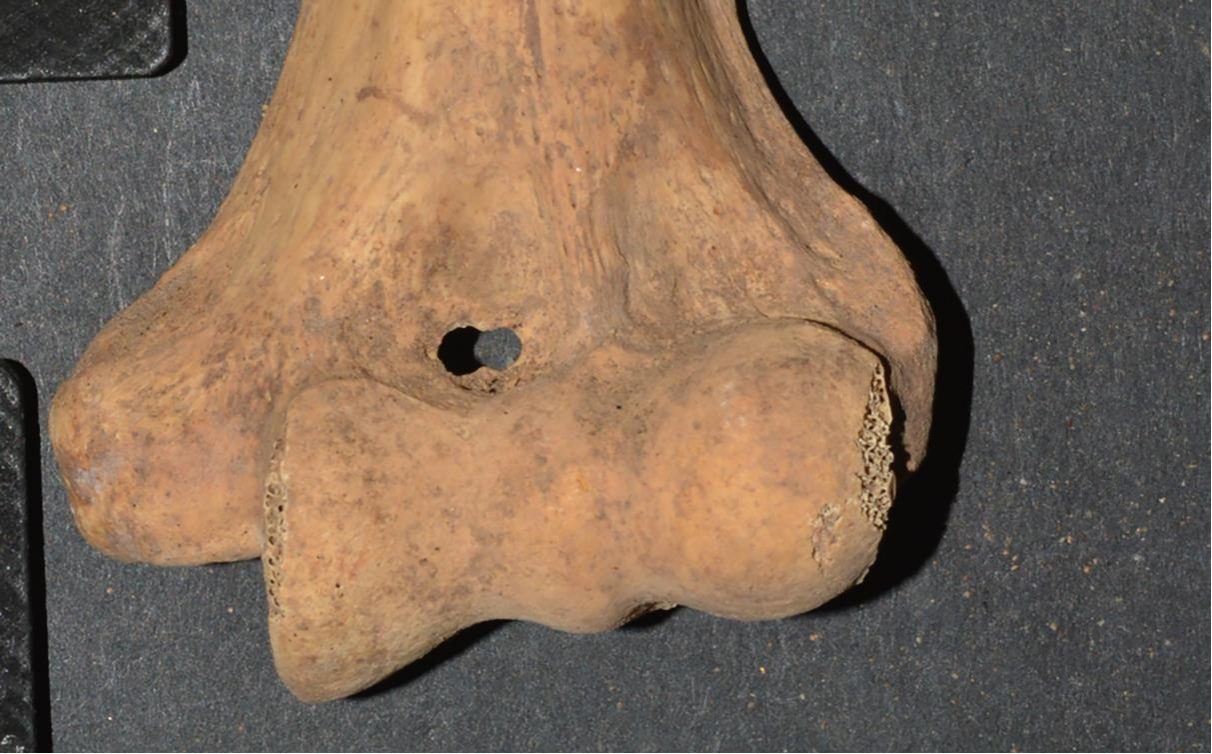
(b)

**Figure:**
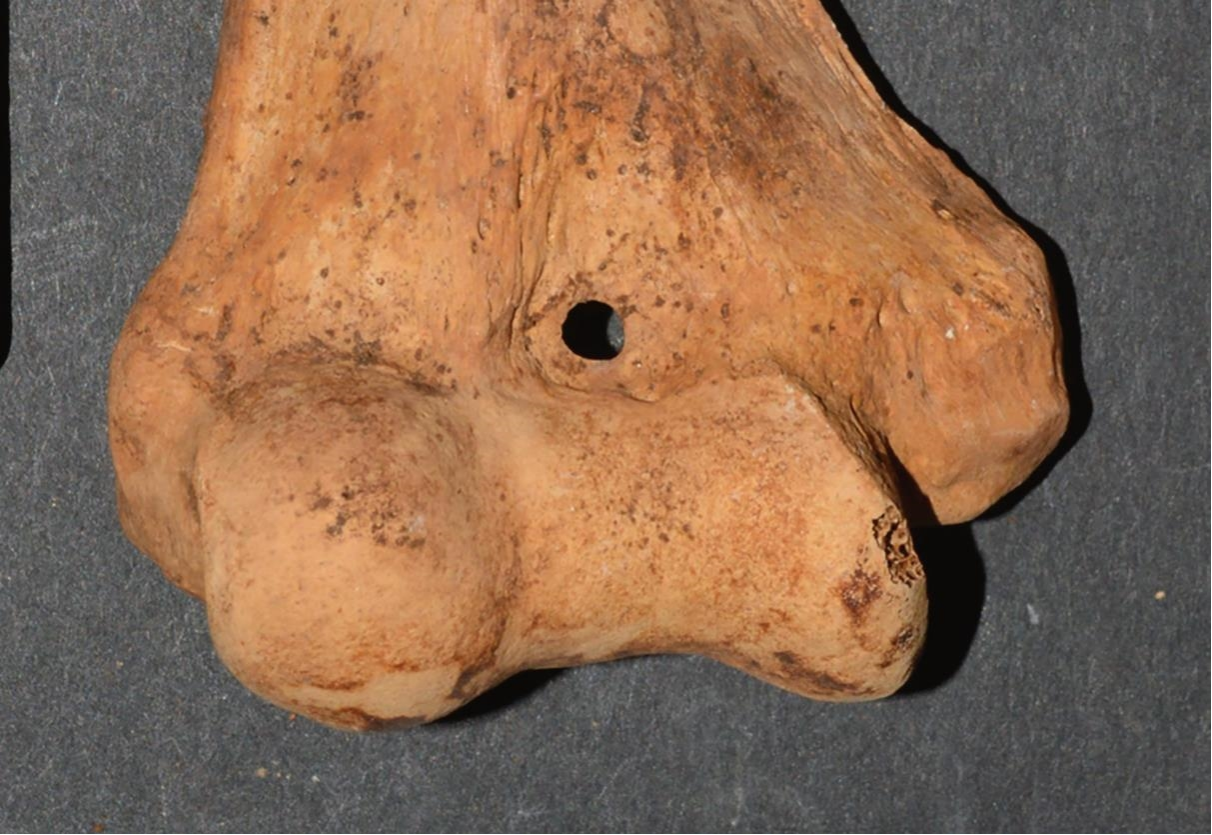
(c)

An analysis was first performed to evaluate for potential differences/bias between the photographic measurements and the caliper measurements. The total epicondylar width was used, as this measurement was available for both the photographic and caliper methods of the distal humerus, the focus of this study. The caliper and photographic measurements were performed by two separate authors, with neither one knowing the results of the other. The average epicondylar width for the caliper and photographic method was 5.86 ± 0.49 and 5.86 ± 0.50 cm, respectively (Student’s *t*‐test, *p* = 0.996). The absolute average difference of the caliper breadth minus the photographic breadth was 0.094 cm, or 1.6% of the average caliper epicondylar width. However, 62 of the differences were ≥ 0 and 66 < 0, indicating neither method was consistently greater than the other. The potential impact of parallax using the photographic method was assessed using the actual difference between the caliper and the photographic method. If a significant parallax effect existed when using the photographic method, the photographic epicondylar width measurements should be consistently greater compared to the caliper measurements; this was not seen. Thus, the caliper method and photographic method give equal results with minimal measurement error (1.6%).

There were many significant differences in humeral dimensions between those with and without an SA (Table 3). For those measurements made with standard archeological calipers, humeri with an SA had smaller epicondylar breadths, vertical humeral head diameters, and humeral shaft diameters in both the ML and anterior/posterior dimensions. There was no difference in humeral length. Measurements from the photographs demonstrated significant differences in TC (which is the same as the epicondylar breadth), condylar articular width, and articular width from the trochlea to the medial aspect of the joint. Again, those with an SA had smaller measurements. There were no differences in the measurements of the anterior (coronoid) fossa width/height and the posterior fossa (olecranon) width and height. The average SA width was 0.70 ± 0.22 cm, and the average height was 0.47 ± 0.14 cm. There were no correlations between the size of the SA and any of the humeral measurements (humeral length, maximum vertical humeral head diameter, maximum width of the humerus at the midshaft in both AP and ML planes, and epicondylar width). The Pearson correlation coefficients between SA width and the six measurements ranged from 0.02 to 0.23 for SA and for SA height from 0.04 to 0.14 (all *p* > 0.2). There were also no significant differences between the three types of SA and any of the humeral measurements (all *p* > 0.2).

**Table 3:** Measurements (in centimeter) of humerus with and without a septal aperture.

	All (*n* = 164)	Septal aperture present	Septal aperture absent	*p* value
Humerus length (*n* = 118)	30.81 ± 1.91	30.68 ± 2.0	30.92 ± 1.85	0.52
Vertical head diameter (*n* = 126)	4.18 ± 0.39	4.08 ± 0.38	4.25 ± 0.39	0.014
Maximum shaft diameter medial/lateral (*n* = 115)	1.85 ± 0.21	1.79 ± 0.18	1.90 ± 0.22	0.006
Maximum shaft diameter anterior/posterior (*n* = 115)	1.90 ± 0.21	1.82 ± 0.18	1.95 ± 0.22	0.0008
Epicondylar breadth (*n* = 130)	5.86 ± 0.49	5.75 ± 0.46	5.94 ± 0.50	0.028
Total condylar width (*n* = 155)	5.85 ± 0.51	5.72 ± 0.48	5.95 ± 0.49	0.004
Total articular width (*n* = 153)	3.78 ± 0.30	3.68 ± 0.28	3.84 ± 0.29	0.0005
Trochlear width (*n* = 155)	2.78 ± 0.47	2.63 ± 0.31	2.88 ± 0.54	0.0002
Olecranon fossa width (*n* = 161)	2.66 ± 0.26	2.65 ± 0.25	1.65 ± 0.22	0.39
Olecranon fossa height (*n* = 161)	1.79 ± 0.30	1/91 0.32	2.13 ± 0.39	0.89
Coronoid fossa width (*n* = 164)	1.66 ± 0.23	1.68 ± 0.22	1.65 ± 0.22	0.31
Coronoid fossa height (*n* = 164)	2.13 ± 0.38	2.14 ± 0.36	2.13 ± 0.39	0.89
Septal aperture width (*n* = 67)	0.70 ± 0.22	0.70 ± 0.22	—	
Septal aperture height (*n* = 67)	0.47 ± 0.14	0.47 ± 0.14	—	

## 4. Discussion

First, certain background information regarding the Tombos site is needed for this discussion. Excavations at Tombos revealed that it was founded as a fortified town by the Egyptian New Kingdom empire ca. 1450 BCE. The associated cemetery began shortly after the founding of the town and is organized into three main cemetery areas. In the north, there are underground chamber tombs that are Egyptian in style and were built for multiple individuals. Based on burial inclusions and practices, it is likely these tomb occupants are “middle‐class” in such roles as lower ranking bureaucrats, scribes, artisans, and servants [29]. The west cemetery consists of Egyptian‐style elite‐built tombs including pyramids, chapels, and shaft tombs. This area contains the burial of high‐level administrators. These substantial tombs likely included an elite individual as well as family members and household staff. There are small individual pit tombs scattered around the north and west cemetery as well [29]. In the east, there are tumulus graves built in styles similar to the local Nubian practices. These local style graves begin during the New Kingdom colonial period and extend through the early Napatan period, after the end of Egyptian control. These tombs usually contained one to three individuals and would have required time and resources to build [23]. Within all of the cemetery areas and tombs, there is evidence of ancient disturbance (reuse and/or looting) that resulted in some commingled remains. Due to preservation issues and disturbance, artifacts and burial containers are not associated with all recovered individuals [29].

Strontium isotope analysis [30] indicates that nonlocal individuals, likely from Egypt based on isotope values and archeological evidence, were buried at Tombos during the New Kingdom period. There are no nonlocal burials after the end of the New Kingdom period. Burial practices and burial inclusions indicate that individuals chose both local and Egyptian mortuary traditions. Artifacts associated with Egyptian religious beliefs are included as well as local artifact styles. Burials using both traditions are found within the same tomb, suggesting that this was a community of immigrants, locals, and their descendants. The strontium isotope values also indicate interaction [29]. Based on carbon isotope data, there does appear to be some dietary variation at Tombos with some individuals eating a more local diet and some eating diets more associated with Egyptian practices. However, due to poor preservation, only a small number of individuals at Tombos could be analyzed [31].

Using entheseal changes (alterations at the points of muscle/ligament attachment to bone) as an indication of broad levels of physical activity, variation in individuals at Tombos is apparent. Previous studies indicated that the individuals from the north cemetery chamber tombs, who comprised “middle‐class” individuals showed very low rates of physical activity [32]. The Eastern cemetery of tumulus graves includes individuals with slightly higher levels of entheseal change, which may be associated with subsistence strategies and granite quarrying [33]. A very recent analysis of the Western cemetery indicates a mix of individuals with varying physical activity levels. Some individuals show the highest levels found at the site; based on burial practices, they are interpreted as low‐status high‐labor staff associated with elite community member households. Due to preservation and ancient disturbance, it is likely that the majority of individuals examined were less elite individuals, as it was often impossible to excavate the chambers where the elite primary tomb owners were buried [34]. In summary, nonlocal individuals, likely from Egypt, were buried at Tombos during the New Kingdom period, and entheseal changes demonstrate variation in physical activity within these individuals.

With this background information, the 41% prevalence of SA we found in the Tombos population is very similar to the 44.5% of Egyptian adults from 1938 to 1759 BCE (Hrdlička) (Table 1). It is lower than the 60% of the El‐Hesa archeological sample from Nubia (42 Nubian individuals from the Late Roman period [347–395 CE] El‐Hesa cemetery) [3] and the 57.2% in ancient Libyans [20] (exact dates not given). It is somewhat higher than the frequencies in prehistoric Native Americans studied by Ferguson and Stewart [19], which ranged from 20% to 37%. For further comparisons, the reader is referred to Table 1.

We noted no difference in SA prevalence by laterality. Of the 67 humeri with an SA, 32 occurred in the right and 35 in the left humerus (*p* = 0.71, one‐way Pearson chi‐square). In prehistoric Native Americans studied by Lamb [18], SA prevalence was 63% in left and 44% in right humeri in those from the Salado I Arizona archeological site, 32% of left, and 7% of right humeri in those from the Zuni New Mexico archeological site, and 37% left and 20% right if multiple mounds. In ancient Libyans, of the 390 humeri with SA, 218 (56%) were left, and 172 (44%) were right humeri [20]. In those from Ostrów Lednicki, Poland, from the 11th to 12th centuries AD, the frequency of SA was almost the same in the left and right humeri (45% and 43%, respectively) [15]. The other archeological studies do not mention SA prevalence by laterality. A meta‐analysis of SA prevalence in modern‐day studies indicates that SA is more common in the left humerus (26.5%) compared to the right humerus (19.4%) [1].

We noted no difference in SA prevalence by the individual’s sex (sex estimated in 113 of the specimens). This appears to be the first archeological study of SA from the BCE, noting SA prevalence by sex. The archeological studies by Lamb [18], Ferguson and Stewart [19], McAlister [20], and Myszka et al. [15] did not separate their findings by sex of the individual. Regarding other archeological studies, Baker et al. [3] noted no differences in SA prevalence by sex in 42 Nubians from the Late Roman period, but there was a difference in 61 Native American humeri from the Archaic period (66% female and 25% male). Hrdlička [4] found the prevalence of SA in 573 ancient Egyptian humeri to be 33% in males and 57% in females. Glanville [10] noted that the prevalence of SA in medieval Dutch populations was 7.3% in female and 4.7% in male humeri. Woo [11] noted that an SA was present in 8.7% of 183 Chinese male humeri and 11% of 109 female humeri. Pietrusewsky et al. [12] in a study of 579 humeri noted a prevalence of SA in 38.6% of females (152 of 394) and 8.6% of males (16 of 185). Mays [14] in a study of 183 humeri noted a prevalence of 1% in males (1 of 104) and 19% in females (15 of 79). Kubicka et al. [16] in a study of 142 humeri described an SA prevalence of 37% in males (20 of 54) and 51% in females (45 of 88). Other archeological studies [2, 5–9, 13] do not have appropriate data from which to determine the SA prevalence by sex. A meta‐analysis of SA prevalence in modern‐day studies indicates that an SA is more common in females (21.9%) compared to males (12.1%) [1]. These differences in the prevalence of SA between/within archeological studies as well as that of modern‐day studies further support a genetic etiology.

Less robust/smaller humeri had a higher prevalence of SA in this study. This appears to be the first archeological study looking at humerus size and the occurrence of SA in individuals from the BCE era. Our results are similar to several modern‐day studies [14, 17, 21, 35, 36]. Trotter [36] noted that the weight of the bones with SAs, especially in Blacks, was less than that of those without SAs. In an English medieval population, Mays [14] found no differences in humerus size between those and without an SA. Mays [14] also suggested that in a larger humerus, the contact of the ulna in hyperextension with the thin bony septum is insufficient to create a perforation with more robust bones preventing this perforation. Mays [14] also stated that their data could not totally support the theory that elbows with larger and more prominent coronoid or olecranon processes were likely to develop SA, refuting the mechanical theory. Our results demonstrated that an increased prevalence of SA in smaller humeri would indirectly support a more mechanical etiology. However, with the well‐known predominance of right‐handedness in humans, the right humerus would be expected to demonstrate a higher prevalence of SA due to increased physical activity in the right elbow compared to the left, if the SA was due to mechanical issues (elbow hyperextension). The lack of differences in the prevalence of SA between right and left humeri and the high prevalence of bilaterality seen in this study lends further support to a genetic etiology rather than a mechanical hyperextension etiology.

The types of SAs in this study were oval/elliptical in 49 (73%), irregular in 10 (15%), and circular in 8 (12%); a present‐day meta‐analysis of 20,338 SAs [1] found that the oval percentage (71.0%) was similar, but the circular percentage was greater (22%) while the irregular percentage was less (3.0%). They also [1] noted that 3.5% were triangular; in this smaller study, we found no triangular SAs. Pires et al. [1] did not discuss why there are different shapes of SAs, nor how they correlated with the size of the humerus. Multiple other authors, while noting differences in SA shape, also did not surmise the reasons for the different shapes. We noted no differences in any of the humeral measurements (humeral length, maximum vertical humeral head diameter, maximum width of the humerus at the midshaft in both AP and ML planes, and epicondylar width) by type of SA. We suggest that these different types of SA simply reflect human biological variation with no particular reason for each type. The average size of the SA in this study had a height of 4.7 mm and a width of 7.0 mm. A meta‐analysis of present‐day humans with SAs [1] found a height of 4.09 mm and a width of 5.06 mm in right humeri and a height of 4.43 mm and a width of 5.45 mm in left humeri; these differences were not statistically significant. While the heights are quite similar between the values of Pires et al. [1], the widths are slightly greater by 2 mm in the Tombos sample. This is, however, likely not a significant difference.

We noted no difference in the prevalence of SA between the New Kingdom and early Napatan periods. Bioarchaeological analyses indicate that physical activities between these two periods may have differed [23]. During the Egyptian colonial New Kingdom period, Tombos inhabitants were part of an administrative center, which would have included government officials and workers, scribes, and artisans. In the postcolonial early Napatan period, transitions likely occurred after the fall of the Egyptian empire; changes in labor activities related to subsistence, construction, and granite quarrying are evident, suggestive of possible heavier physical labor. The New Kingdom sample available for the study of activity patterns primarily comes from a “middle‐class” segment of the population while the early Napatan sample may also include some elite individuals evidenced by elaborate burial goods [23]. More recent analysis of activity patterns on a larger sample from Tombos indicates that some individuals buried in the New Kingdom Western cemetery were also engaging in heavy labor and may have been nonelite staff working in elite households [34]. Since there is possible variation in activity levels across the site, this gives further support to the etiology of SA being genetic rather than other reasons (mechanical or activity related), leading into the next points.

The etiology of SA is unknown, with mechanical, bone robusticity, genetic, environmental, and a combination of factors all being suggested as possible causes [4, 6, 7, 9, 10, 13, 14, 16, 17, 20, 21]. The mechanical hypothesis states that hyperextension of the elbow causes a mechanical perforation [20]. With increasing human age, it would be expected to see an increasing percentage of SA, but that has not been seen [17]. Rather, the opposite has been noted, with SA being more prevalent in younger adults compared to older adults. Myszka [17] also noted that looser triceps lead to greater looseness of the elbow, greater protrusion of the olecranon process, and consequent SA formation. In the Tombos population, the highest prevalence of SA was in the 15–24‐year age group, suggesting that it is not truly mechanical. Based on entheseal changes, young adults generally have the lowest levels of physical activity, while the higher levels are generally composed of older adults [34]—further evidence to refute this mechanical hypothesis. Additionally, our results demonstrated no difference in the prevalence of SA by laterality, which also refutes the mechanical hypothesis. Upper extremity laterality is the right‐hand dominant in the majority of the population, and if physical activity levels are an indirect proxy for mechanical etiology, then a major difference between right and left SA prevalence should have been seen, with it being more prominent in the right upper extremity. This was not observed.

Another theory involves bone robusticity. Mays [14] stated that SAs were more common in females and in left bones, leading early investigators to suggest that they were more frequent in “weaker” bones. However, subsequent literature offered little support for the concept that a relationship between humeral gracility and SA exists in humans beyond any relationships with sex and laterality. Ndou and Schepartz [21] noted that smaller individuals were prone to the formation of the SA. Females in their study had a higher percentage of SAs as well as smaller humeri determined by humeral length, humeral head circumference, midshaft humeral circumference, and humeral epicondylar breadth. Ndou and Schepartz [21] noted that a significantly longer olecranon process was associated with an SA for both males and females, even though the olecranon process on average was longer in males than in females. Mays [14] suggested that in a larger humerus, the contact of the ulna in hyperextension with the thin bony septum is insufficient to create a perforation, with more robust bones preventing this perforation. In the Tombos sample, we confirmed that SA was more common in humeri with smaller head diameters, epicondylar breadth, and midshaft size.

A third hypothesis is that it is genetic. A genetic etiology can explain the differences seen in different ancestral populations but does not explain why it may be less common in older adults compared to younger adults. The genetic make‐up of the Tombos sample is a combination of people who originated in Egypt and Nubia, but it is still difficult to compare with the few archeological studies of SA from northern Africa and the mid‐East [4, 5, 8, 20] as these are different populations from different eras. However, a high prevalence of bilaterality is often interpreted to support a genetic etiology [6, 7, 9, 13, 37]. Anderson [9] described nonmetric, discrete traits as anomalies in normal skeletal anatomy and simply recorded them as present or absent, with most being thought to have a genetic origin. Anderson supported this statement with several references of nonmetric traits aside from SA [38–42]. Similarly, Cavichi et al. [37] studied the SA and its relationship with humeral and ulnar measurements and suggested a genetic association for SA using multivariate analysis of multiple humeral measurements between males and females and those with and without SA. For the other studies, the overall prevalence of SA was 33% (18 of 55) in the study of Taxman [6], 15.7% (45 of 287) in the study of Oláh [13], and ranged from 16% to 26% in the study of Nagar [7]. These authors believe that SA is genetic due to their high prevalence of SA as a nonmetric trait. The high percentage of bilaterality in intact individuals with both right and left humeri available in this present study was 81%, even higher than many of the above studies, which is thus further supportive of a genetic etiology.

Finally, other investigators believe that the etiology of SA is multifactorial [4, 10, 16, 17]. These factors are genetic, race/ethnicity, bone robusticity, ligamentous/muscular laxity, and mechanical (e.g., elbow range of motion with associated hyperflexion/extension and ulno‐humeral shape differences). Glanville [10] believes that SA is due to a combination of mechanical, genetic, and environmental factors and that bilateral, sexual, and population differences in SA frequency are associated with differences in the elbow joint range of motion. Kubicka et al. [16] believe that SA is due to both genetic and mechanical factors. Hrdlička [4] primarily believes that it is a combination of mechanical and genetic factors. While it is likely that some cases of SA may be multifactorial, in the Tombos population, genetics seems to be the most likely etiology due to the high prevalence of bilaterality. Of course, the elbow range of motion/muscular laxity cannot be truly ascertained for this ancient collection, and thus, there may be a certain component of a mechanical etiology, especially in view of our findings regarding increased SA in more gracile/less robust specimens.

### 4.1. Limitations of the Study

In archeological collections, only the individuals available can be studied, and it is not truly known if this Tombos sample is a statistically accurate sampling of the Tombos population during those time periods. While we did not perform intra‐ and interobserver variability measurements in this study, we did analyze if there were any differences between the maximum epicondylar breadth determined by the anthropology author using calibers to those of the total anterior width obtained from the photographs. These measurements were performed by two different authors, with neither one knowing the other author’s measurements. The results between the epicondylar breadth and TC are strikingly similar (Table 3), and upon statistical analysis, there was no significant difference between the two (*p* = 0.996), indicating nearly complete agreement. We thus conclude that the measurements in this study are accurate.

## 5. Conclusion

In this study of 164 ancient Nubian humeri of acceptable quality (from 240 humeri reviewed) from Tombos, an SA was present in 40.9% with no differences by era, sex, age group, right versus left humeri, individual versus commingled burial, and complete versus incomplete humeri. The shapes of the apertures were oval/elliptical in 49 (73%), irregular in 10 (15%), and circular in 8 (12%). Those with an SA were less robust as shown by smaller epicondylar breadths and humeral head and shaft diameters. There were no differences in the measurements of the anterior (coronoid) fossa width/height and the posterior fossa (olecranon) width and height. In the 16 individuals with paired humeri and SAs, the SA was bilateral in 13 individuals, left humerus in 2 individuals, and right humerus in 1 individual. This 81% prevalence of bilaterality is supportive of a strong genetic component in the etiology of SA in this population.

## Author Contributions

**Jenessa Love:** data curation, investigation, methodology, validation, visualization, writing – original draft, and writing – review and editing. **Ashlyn J. Morris:** data curation, formal analysis, investigation, methodology, validation, visualization, writing – original draft, and writing – review and editing. **Heidi Joelle Althaus:** data curation, formal analysis, investigation, methodology, validation, visualization, writing – original draft, and writing – review and editing. **Faith Kylee Darden:** data curation, formal analysis, investigation, methodology, validation, visualization, writing – original draft, and writing – review and editing. **Michele R. Buzon:** conceptualization, data curation, funding acquisition, investigation, methodology, project administration, resources, supervision, validation, visualization, writing – original draft, and writing – review and editing. **John E. Kuhn:** conceptualization, methodology, visualization, writing – original draft, and writing – review and editing. **Randall T. Loder:** conceptualization, data curation, formal analysis, investigation, methodology, project administration, resources, supervision, validation, visualization, writing – original draft, and writing – review and editing.

## Funding

The study was funded by National Geographic Society (10.13039/100006363) and National Science Foundation (10.13039/100000001; BCS‐0917824, BCS‐0907815, and BCS‐0313247).

## Disclosure

All authors have read and approved the final version of the manuscript. Randall T. Loder had full access to all of the data in this study and takes complete responsibility for the integrity of the data and the accuracy of the data analysis.

## Conflicts of Interest

R.T.L. is a member of the editorial board for the *Journal of Pediatric Orthopaedics*, the *Journal of Children’s Orthopaedics* and *Children* (*Basel*). He receives royalties from publishers of book chapters not related to the subject of this manuscript. M.R.B. is a board member and officer of the American Sudanese Archaeological Research Center and an editorial board member of the *Journal of the American Research Center in Egypt* and the Brepols Publisher book series *Nubia: Studies in Archaeology and History of Northeast Africa*. She receives honoraria for lectures. J.E.K. is the editor‐in‐chief of the *Journal of Shoulder and Elbow Surgery* for which he receives compensation. J.L., A.J.M., H.J.A., and F.K.D. have no conflicts of interest.

## Data Availability

The data is available upon reasonable request to the corresponding author. Access to the archeological skeletal remains can be requested by contacting the curator, Dr. Michele Buzon, at Purdue University.
